# Using Indigenous Standards to Implement the CARE Principles: Setting Expectations through Tribal Research Codes

**DOI:** 10.3389/fgene.2022.823309

**Published:** 2022-03-21

**Authors:** Stephanie Russo Carroll, Ibrahim Garba, Rebecca Plevel, Desi Small-Rodriguez, Vanessa Y. Hiratsuka, Maui Hudson, Nanibaa’ A. Garrison

**Affiliations:** ^1^ Mel and Enid Zuckerman College of Public Health, University of Arizona, Tucson, AZ, United States; ^2^ Native Nations Institute, Udall Center for Studies in Public Policy, University of Arizona, Tucson, AZ, United States; ^3^ Library and Information Sciences, School of Information, University of Arizona, Tucson, AZ, United States; ^4^ Law Library, School of Law, University of South Carolina, Columbia, SC, United States; ^5^ Department of Sociology, College of Social Sciences, University of California, Los Angeles, Los Angeles, CA, United States; ^6^ American Indian Studies Program, College of Social Sciences, University of California, Los Angeles, Los Angeles, CA, United States; ^7^ Center for Human Development, College of Health, University of Alaska Anchorage, Anchorage, AK, United States; ^8^ Te Kotahi Research Institute, University of Waikato, Hamilton, New Zealand; ^9^ Institute for Society and Genetics, University of California, Los Angeles, Los Angeles, CA, United States; ^10^ Institute for Precision Health, David Geffen School of Medicine, University of California, Los Angeles, Los Angeles, CA, United States; ^11^ Division of General Internal Medicine and Health Services Research, David Geffen School of Medicine, University of California, Los Angeles, Los Angeles, CA, United States

**Keywords:** genetic research, Indigenous, data sovereignty, data governance, CARE principles

## Abstract

Biomedical data are now organized in large-scale databases allowing researchers worldwide to access and utilize the data for new projects. As new technologies generate even larger amounts of data, data governance and data management are becoming pressing challenges. The FAIR principles (Findable, Accessible, Interoperable, and Reusable) were developed to facilitate data sharing. However, the Indigenous Data Sovereignty movement advocates for greater Indigenous control and oversight in order to share data on Indigenous Peoples’ terms. This is especially true in the context of genetic research where Indigenous Peoples historically have been unethically exploited in the name of science. This article outlines the relationship between sovereignty and ethics in the context of data to describe the collective rights that Indigenous Peoples assert to increase control over their biomedical data. Then drawing on the CARE Principles for Indigenous Data Governance (Collective benefit, Authority to control, Responsibility, and Ethics), we explore how standards already set by Native nations in the United States, such as tribal research codes, provide direction for implementation of the CARE Principles to complement FAIR. A broader approach to policy and procedure regarding tribal participation in biomedical research is required and we make recommendations for tribes, institutions, and ethical practice.

## Introduction

As technological advances have generated immense amounts of biomedical data, the Indigenous Data Sovereignty (IDSov) movement has emerged to exert stronger control and oversight over data generated from Indigenous Peoples. Once subject to localized systems of management, biomedical data are now organized and stored in large-scale databases, allowing researchers worldwide to access and utilize data for new analyses. The governance of large-scale databases, many of which adopt broad data sharing models, often stands in contrast with stricter mechanisms of protection and relationships of trust that facilitated the original data collection. This disconnect is clearly evident in the case of Indigenous communities who have often challenged the extractive nature of genetic research (Boyer et al., 2007; Shaw et al., 2013; Trinidad et al., 2015; Haring et al., 2018; Chadwick et al., 2019; Dirks et al., 2019). We support the call for more open, inclusive, and equitable participation in research and innovation to resolve the tension between openness and innovation, on the one hand, and Indigenous rights and interests, on the other. This is a tension that pervades the current discourse on genetic diversity (Hudson et al., 2020; Welch et al., 2021).

Historically, biomedical data may not have been collected or utilized in ways that align with community rights and interests. The results are research with little or no benefit to the communities from which the data originated, potential biases in data interpretation, dwindling participation in genetics and genomics research, and limited oversight by the people from whom the data are collected (Garrison et al., 2019a). These negative experiences compound as biomedical and data futures move towards big data and large-scale biobanking. At the same time, the resurgence of Indigenous self-determination and the advancement of IDSov prompts a reexamination of data governance (Kukutai and Taylor 2016a; Garrison et al., 2019a; Carroll et al., 2020; Hudson et al., 2020; Walter et al., 2021). At a fundamental level, IDSov articulates the rights of Indigenous Peoples and nations to govern the collection, application, and use of data about their peoples, communities, lands, and resources (Kukutai and Taylor, 2016b).

This article outlines the relationship between sovereignty and ethics in the context of data to describe the collective rights that Indigenous Peoples assert to increase control over their biomedical data. Then drawing on the CARE Principles for Indigenous Data Governance (Collective benefit, Authority to control, Responsibility, and Ethics), we explore how standards already set by Native nations, such as tribal research codes, provide direction for implementing the CARE Principles. We close with recommendations for using tribal codes, laws, policy documents, and protocols to operationalize the CARE Principles as a way to spur translational genetics research that benefits Native nations, as well as rural and urban Indigenous communities.

### Indigenous Peoples and Data

For the purposes of this paper, we define Indigenous Peoples in the US as American Indian, Alaska Native, Native Hawaiian, and other communities who are indigenous to the US and its territories. We will use Native nations and tribes interchangeably to refer to tribal nations in the US. The federal government recognizes 574 tribes in the US as sovereign nations with their own legal and political structures to govern their citizens and homelands (Department of the Interior, 2021). In addition, many other Indigenous Peoples exert sovereignty as state-recognized (National Conference of State Legislatures, 2019) or un-recognized nations, including those in the state of Hawai’i and US territories. Sovereignty refers to the collective powers of a nation, such as the power to grant access to the population or to negotiate treaties between nations. As sovereign nations, tribes have the power to govern via their own structures, determine their own citizenship, and regulate tribal business (Duthu, 2008).

Indigenous Peoples have always been “researchers,” demonstrated by their collecting, analyzing, and managing data for decision-making, knowledge transfer, and other uses. Historical and ongoing colonialism disrupted, co-opted, and suppressed Indigenous research methodologies and methods (Smith, 2012). Indigenous data, whether born digital or not, include information, knowledge, specimens, and belongings about Indigenous Peoples to which they relate at both the individual and collective levels (Carroll et al., 2020; Rainie et al., 2019; Lovett et al., 2019). IDSov returns authority over data about Indigenous nations and their citizens, communities, and resources (wherever they may be located) back to the tribes from whom the data derive (Kukutai and Taylor, 2016b). Indigenous Data Governance (IDGov) enables tribal ways of knowing and doing to guide Indigenous decision-making; it is a practical expression of IDSov (Rainie et al., 2017; Maiam nayri Wingara, 2018).

Increasingly over the past 50 years, tribes in the US have developed policies and procedures for the oversight of research within their nations’ physical jurisdiction and beyond tribal lands. Other Native nations rely on tribal colleges, tribal organizations, or the Indian Health Service to provide research oversight on their behalf (Around Him et al., 2019). Federally-recognized tribes are in the strongest legal position to assert authority over their data (Tsosie, 2019). Non-federally-recognized tribes and Indigenous Peoples worldwide experience numerous issues in exercising rights over their data that may be different from federally-recognized tribes (Kukutai and Taylor, 2016a; Walter et al., 2021). However, we posit that learnings from federally-recognized tribes’ codes can broadly benefit Indigenous Peoples as they implement laws, policies, and practices to govern their data and research.

IDGov and tribal research governance complement one another: some data are research data that are subject to both data governance and research governance. Thus, Indigenous research governance becomes a mechanism for enhancing IDGov as tribes assert IDSov.

### Indigenous Peoples’ Increased Oversight of Biomedical Research

IDSov requires heightened consideration in projects that evoke a government-to-government relationship, such as federally funded projects that seek to recruit large numbers of Indigenous Peoples nationwide. In these cases, strong relationships and effective data governance systems at the tribal level are paramount for ensuring equitable participation in federally funded research and culturally rigorous results. At the same time, non-tribal institutional policies and practices must also evolve to promote and protect the sovereign rights and interests of Indigenous Peoples.

American Indian and Alaska Native populations are not simply ethnic or racial groups, nor are they vulnerable or “special” populations. Tribes maintain a unique political status and confer citizenship just like other nation states. Tribal citizenship persists regardless of residence on or off tribal lands. Also called tribal enrollment, tribal citizenship is not the same as self-identification nor is it the same as genetic ancestry (Tallbear, 2013). Tribal citizenship is a political designation similar to US citizenship. This political designation is the foundation for IDSov. Yet, the inclusion of Indigenous people off tribal lands challenges the reach of tribal oversight of research over enrolled tribal citizens. Approximately 78% of self-identified American Indian and Alaska Native individuals live off tribal lands, and approximately 60% primarily reside in urban areas (Norris et al., 2012). For Indigenous people living off tribal lands, questions arise regarding how tribes will govern information about them when data are collected and reside outside the jurisdictional boundaries of the tribal nation. Additional questions include how other institutions, such as intertribal non-profit organizations and universities, will steward and protect data about Indigenous Peoples and individuals.

The recognition of IDSov by federal agencies and large repositories funded by organizations like the National Institutes of Health (NIH) is an important first step. The use of already existing tribal expectations delineated in reports, policies, and practices are important next steps to align federal programs with tribal rights and expectations via IDSov (Tribal Collaboration Working Group, 2018). In late 2019, the National Congress of American Indians (NCAI) (National Congress of American Indians, 2019a) asserted that even in the absence of formal tribal approval processes, researchers must establish a process to obtain approval that allows for tribal oversight of tribal data. Furthermore, the NCAI membership passed Resolution ABQ-19-061 that “calls on NIH to consult with tribal nations, provide a process for tribal nations to have oversight over any data and biospecimens from their tribal citizens, and restrict use of data associated with tribal nations until tribal oversight is in place” (National Congress of American Indians, 2019b). This resulted in developing a formal tribal consultation process (National Congress of American Indians, 2021; Haozous et al., 2021).

Tribal concerns about data use and data sharing have generated many discussions in federal agencies, universities, professional societies, and Indigenous communities. To build ethical university-tribal partnerships, it is necessary to recognize tribes as sovereign nations, acknowledge tribal intellectual property, and respect tribal data sharing preferences (James et al., 2014). Indigenous individuals’ concerns about privacy and confidentiality also extend to promotion of tribal rights to control data and protection of collective tribal confidentiality and privacy in data and research (Taitingfong et al., 2020). In interviews with Indigenous leaders, scholars, and tribal research review members, support for tribal oversight of data is seen as a viable solution to the challenges of data access, management, and sharing (Garrison et al., 2019b). Given the history of exploitative research with tribal communities, the ability of tribes to review inaccurate, harmful, or stigmatizing information before publication or distribution is crucial both to preventing the misuse of their data and to supporting sound scientific practice (Garrison et al., 2019a). This is increasingly important as biomedical and genomics research moves toward broad data sharing policies.

Indigenous data oversight has increased in response to support of broad data sharing by funders and scientists. The NIH Genomic Data Sharing policy requires federally-funded investigators to deposit de-identified data into federal databases to promote secondary analyses (National Institutes of Health, 2014). However, the policy allows a data sharing exception that recognizes some tribal laws may not permit broad data sharing (Hiratsuka et al., 2020). Some tribal laws and policies dictate that all data generated from a research study is property of the tribe and all data must be returned to the tribe at the conclusion of the study. A resulting concern about the data sharing policy is that the allowable exceptions are not clearly understood or recognized by all researchers, institutions, or journal editors. For example, some investigators who conduct research with Indigenous communities have been asked by journal editors to submit the data to federal databases, even when the agreement with the tribe is not to share data.

## Implementation of CARE Principles Guided by Tribal Oversight

The current structures that are in place for federal biomedical data governance, in particular the Common Rule (Office of Human Research Protections, 2017), fail to align with the rights and interests of Indigenous nations and communities (Hudson et al., 2020). Rather than demanding that representatives of Indigenous communities participate in these existing governance structures, we argue for sovereign control—that is, Indigenous nations controlling ownership, governing storage, and dictating parameters for data use and reuse. We also promote policy innovations for other institutions that both adhere to tribal sovereignty and protect Indigenous people living off tribal lands or who self-identify as Indigenous (i.e., not tribally affiliated).

This section introduces the CARE Principles for Indigenous Data Governance as high-level guidance for enhancing IDSov in research and data governance. This section also examines the sovereign expectations that tribes set for researchers and institutions to support Indigenous Peoples’ efforts to reclaim control and oversight of data, including biospecimens.

### The CARE Principles for Indigenous Data Governance

The CARE Principles define Collective benefit, Authority to control, Responsibility, and Ethics, and their relationship to engagement with and for secondary use of Indigenous data (Research Data Alliance Interest Group, 2019). The CARE Principles and the sub-principles (see Table 1) enhance and extend the ‘FAIR Principles’ for scientific data management (Findable, Accessible, Interoperable, Reusable; Wilkinson et al., 2016) by centering equity and ethics as core guiding principles alongside those set out by FAIR. The CARE Principles reflect the crucial role of data in advancing Indigenous innovation and self-determination by focusing on people and purpose-oriented standards to be used alongside mainstream data guidelines (Carroll et al., 2020).

**TABLE 1:** The CARE Principles for Indigenous Data Governance and sub-principles.

COLLECTIVE BENEFIT: Data ecosystems shall be designed and function in ways that enable Indigenous Peoples to derive benefit from the data
C1: For Inclusive Development and Innovation
Governments and institutions must actively support the use and reuse of data by Indigenous nations and communities by facilitating the establishment of the foundations for Indigenous innovation, value generation, and the promotion of local self-determined development processes
C2: For Improved Governance and Citizen Engagement
Data enrich the planning, implementation, and evaluation processes that support the service and policy needs of Indigenous communities. Data also enable better engagement between citizens, institutions, and governments to improve decision-making. Ethical use of open data has the capacity to improve transparency and decision-making by providing Indigenous nations and communities with a better understanding of their peoples, territories, and resources. It similarly can provide greater insight into third-party policies and programs affecting Indigenous Peoples
C3: For Equitable Outcomes
Indigenous data are grounded in community values, which extend to society at large. Any value created from Indigenous data should benefit Indigenous communities in an equitable manner and contribute to Indigenous aspirations for wellbeing
AUTHORITY TO CONTROL: Indigenous Peoples’ rights and interests in Indigenous data must be recognised and their authority to control such data be empowered. Indigenous data governance enables Indigenous Peoples and governing bodies to determine how Indigenous Peoples, as well as Indigenous lands, territories, resources, knowledges and geographical indicators, are represented and identified within data
A1: Recognizing Rights and Interests
Indigenous Peoples have rights and interests in both Indigenous Knowledge and Indigenous data. Indigenous Peoples have collective and individual rights to free, prior, and informed consent in the collection and use of such data, including the development of data policies and protocols for collection
A2: Data for Governance
Indigenous Peoples have the right to data that are relevant to their world views and empower self-determination and effective self-governance. Indigenous data must be made available and accessible to Indigenous nations and communities in order to support Indigenous governance
A3: Governance of Data
Indigenous Peoples have the right to develop cultural governance protocols for Indigenous data and be active leaders in the stewardship of, and access to, Indigenous data especially in the context of Indigenous Knowledge
RESPONSIBILITY: Those working with Indigenous data have a responsibility to share how those data are used to support Indigenous Peoples’ self-determination and collective benefit. Accountability requires meaningful and openly available evidence of these efforts and the benefits accruing to Indigenous Peoples
R1: For Positive Relationships
Indigenous data use is unviable unless linked to relationships built on respect, reciprocity, trust, and mutual understanding, as defined by the Indigenous Peoples to whom those data relate. Those working with Indigenous data are responsible for ensuring that the creation, interpretation, and use of those data uphold, or are respectful of, the dignity of Indigenous nations and communities
R2: For Expanding Capability and Capacity
Use of Indigenous data invokes a reciprocal responsibility to enhance data literacy within Indigenous communities and to support the development of an Indigenous data workforce and digital infrastructure to enable the creation, collection, management, security, governance, and application of data
R3: For Indigenous Languages and Worldviews
Resources must be provided to generate data grounded in the languages, worldviews, and lived experiences (including values and principles) of Indigenous Peoples
ETHICS: Indigenous Peoples’ rights and wellbeing should be the primary concern at all stages of the data life cycle and across the data ecosystem
E1: For Minimizing Harm and Maximizing Benefit
Ethical data are data that do not stigmatize or portray Indigenous Peoples, cultures, or knowledges in terms of deficit. Ethical data are collected and used in ways that align with Indigenous ethical frameworks and with rights affirmed in UNDRIP. Assessing ethical benefits and harms should be done from the perspective of the Indigenous Peoples, nations, or communities to whom the data relate
E2: For Justice
Ethical processes address imbalances in power, resources, and how these affect the expression of Indigenous rights and human rights. Ethical processes must include representation from relevant Indigenous communities
E3: For Future Use
Data governance should take into account the potential future use and future harm based on ethical frameworks grounded in the values and principles of the relevant Indigenous community. Metadata should acknowledge the provenance and purpose and any limitations or obligations in secondary use inclusive of issues of consent

### Tribal Research Governance as Expectations

The CARE Principles are in the early stages of implementation, with some entities leading the way by collaborating with the Global Indigenous Data Alliance (GIDA) to operationalize the principles within repositories, national ethics frameworks, and United Nations open science guidance (Australian Institute of Aboriginal and Torres Strait Islander Studies, 2020; Carroll et al., 2021; United Nations Educational Scientific and Cultural Organization, 2021; Welch et al., 2021). Large international genomics consortia are already implementing the FAIR principles, but to truly engage and demonstrate respect for marginalized, impacted, excluded, underserved populations, the CARE Principles must be integrated across institutional policies and practices (Wilkinson et al., 2016; Carroll et al., 2021). We draw on federal- and state-recognized Native nations’ research regulations (Table 2) to illustrate how these official documents’ assertions of IDSov set tribal expectations for enacting the CARE Principles*.* Tribal expectations include alignment with tribal priorities, recognizing the locus of control for tribal data, supporting respectful relationships, and addressing inequities in research.

**TABLE 2:** The CARE Principles for Indigenous Data Governance: Tribal expectations that guide implementation.

Principle/Sub-principle	Quotes from Tribal Documents	Tribal Expectations
COLLECTIVE BENEFIT: Data ecosystems, including research life cycle, to be organized in ways open to collective Indigenous input and accessible for collective Indigenous benefit		
C1: For Inclusive Development and Innovation	Researchers shall provide for Tribal oversight of projects and report regularly to the Tribal Council and liaison department of project progress and results. Confederated Tribes of Siletz Indians, (2005)	Project outcomes to align with tribal needs and tribal input to be incorporated into research process
	The tribe will only support community engaged research practices, which requires a high level of collaboration with Cherokee Nation (integrating the ideas of the tribal into the study) and must address Cherokee needs to benefit the citizens. Cherokee Nation, (2019b)	
C2: For Improved Governance and Citizen Engagement	Research should not be conducted until there has been full consultation with all potentially affected communities and individuals including all human research subjects, and each such community and individual have approved the research after full disclosure. Turtle Mountain Band of Chippewa Indians, (2014)	Obligation to engage, consult, and seek approval of both individuals and communities potentially affected by the research
	Researchers are advised to budget funding...to provide adequate resources to cover community education and outreach efforts. Mohawk Nation of Akwesasne, (1996)	
C3: For Equitable Outcomes	Expected benefits of the proposed research, primary or secondary findings, including immediate and long range benefits to... the Nation; the Indian people generally; and society generally. Ho-Chunk Nation, (2005)	Benefits may apply broadly but such benefits must have specific connections to tribal needs and priorities
	Just compensation or fair return includes but is not limited to: obtaining copies of the research findings, authorship, co-authorship or acknowledgment, royalties, fair monetary compensation, copyright, patent, trademark. Mohawk Nation of Akwesasne, (1996)	
AUTHORITY TO CONTROL: Recognition of Indigenous rights regarding research materials and data involve return of findings to community and control of uses outside tribal territory		
A1: Recognizing Rights and Interests	Principle of Prior Rights: This principle recognizes that Indigenous peoples, traditional societies, and local communities have prior, proprietary rights and interests over all air, land, and waterways, and the natural resources within their territories that they have traditionally inhabited or used, together with all knowledge and intellectual property and traditional resource rights associated with such resources and their use. Turtle Mountain Band of Chippewa Indians, (2014)	Tribal claims to ownership of research materials and data, and expressions of prior Indigenous rights to lands, waterways, and natural resources
	This Code shall apply to all research (as defined elsewhere in this Code) conducted within the Nation’s Territory, whether involving human subjects or not, and all research regarding materials wherever located as to which the Nation has a claim of intellectual, cultural or other ownership, legal or equitable. Ho-Chunk Nation, (2005)	
A2: Data for Governance	The process of developing community-based and culturally relevant research should directly include the tribe from the studies inception and supports a tribal agenda (plus whenever possible include local Native American investigators). Cherokee Nation, (2019b)	Findings from research to be returned to the community to support governance and self-determination
	At a minimum, the following information shall be provided by a Medical and Health Care applicant researcher ... (G) ... opportunity for the Community, Districts, and individuals, as appropriate to have periodic reports on the progress of the Medical Health Care Research and to comment on periodic and draft final reports. Gila River Indian Community, (2009)	
A3: Governance of Data	Research information and data generated by and about Navajo individuals, communities, culture represent inalienable intellectual properties of the Navajo people and over which the Navajo Nation will provide oversight. Navajo Nation, (2002)	Tribal governments have right and responsibility to ensure research data used in ways consistent with community values, interests, and priorities
	This principle recognizes that the Tribe and any human research subjects, at its/their sole discretion, have the right to exclude from publication and/or to have kept confidential, any information including information concerning themselves, their health, or their culture, traditional knowledge, traditions, mythologies, or spiritual beliefs ... Three Affiliated Tribes (n.d.)	
RESPONSIBILITY: Researchers to respect Indigenous classifications, restrictions, and practices in relation to data and to advance community’s capacity to manage own data by involving members in research activities		
R1: For Positive Relationships	This principle recognizes the necessity for researchers to respect the integrity, morality, and spirituality of the culture, traditions, and relationships of Tribal members with the world, and to avoid the imposition of external conceptions and standards. Turtle Mountain Band of Chippewa Indians, (2014)	Mutual understanding and respect crucial in engaging Indigenous data, especially those data considered sacred or culturally significant
	Cultural sensitivity training for the researchers as well as research awareness presentations on the Reservation will help develop a mutual understanding in conducting the research projects. Three Affiliated Tribes (n.d.)	
R2: For Expanding Capability and Capacity	The Research Advisory Committee will help to ensure that the proposed research... empowers those involved through education, training and/or authorship. Mohawk Nation of Akwesasne, (1996)	Researchers to strengthen community’s ability to manage own data through training and employment opportunities in research projects
	… Provisions for Native and local preference in employment in all phases of the project, including both on and off Reservation phases. White Earth Nation, (2018)	
R3: For Indigenous Languages and Worldviews	Further, the Karuk Tribe asserts its age-old tradition of reserving domains of knowledge for rightful and culturally appropriate owners, as well as restricting access to this knowledge during certain chronological periods as dictated by time honored Karuk Law. Karuk Tribe, (2015)“Human Subject” means a living or nonliving individual (including human remains) about whom a researcher conducting research obtains information or data through interaction with the individual, involving physical procedures by which data are gathered (for example, blood draws), and/or manipulations of the subject or the subject’s environment. Tohono O’odham Nation (2013)	Recognition and inclusion of Indigenous data norms and practices throughout research process
ETHICS: Obligation to minimize risks and maximize community benefits throughout research life cycle and also to strengthen Indigenous rights by addressing power and other imbalances		
E1: For Minimizing Harm and Maximizing Benefit	Beneficence is not met, no matter how minimal the risks, when there is no maximized benefit to the tribe or its participants. This in turn can lead to an injustice if the benefits gained by that research are denied to the tribe and/or its citizens. Cherokee Nation, (2019a)	Cultural harm to be prevented in research and maximization of benefits to be treated as core rather than incidental aspect of research
	The Legislature also has a fundamental responsibility to protect and preserve the culture of the Nation and to ensure that the IRB permitted activities are conducted in a way that does no harm to the culture of the Nation. Ho-Chunk Nation, (2005)	
E2: For Justice	Both the researcher(s) and Tribe must bring equity to any research contract, agreement, or understanding. This includes finances, community knowledge, networks, personnel, and political or social power. Three Affiliated Tribes (n.d.)	Unequal relations in Indigenous research to be acknowledged and joint efforts to be made by researchers and tribes to address inequities through sharing of power, people, knowledge, and resources
	Community knowledge, networks, and personnel and political or social power are other forms of equity useful to a project. Each of these commodities has value and must be shared between the researchers and the Tribe if a proper agreement is to be formulated. Turtle Mountain Band of Chippewa Indians, (2014)	
E3: For Future Use	At a minimum, the following information shall be provided by an applicant researcher... whether secondary use of any retained specimens is contemplated; informed consent regarding saved specimens and future uses... Ho-Chunk Nation, (2005)	Disclosure, consent, and control required with respect to secondary uses of research materials and data
	What control will the Community or Medical and Health Care Research participants have over the current and future use of the data, and how will the control be exercised?... What control will the Community have over the current and future use of the human biological material, and how will the control be exercised? (9.107) Gila River Indian Community, (2009)	

## Discussion and Recommendations

Below we make recommendations for tribes, other institutions, and ethical practices that leverage Native nations’ codes as standards for researchers and data stewards as they implement the CARE Principles.

### Tribal Law and Policy

Native nations are increasingly using tribal codes to set standards and expectations, exerting their jurisdiction over data, interests, places, and issues both on and off reservations (National Congress of American Indians, 2019a; Hiraldo et al., 2020). Here we share some of the ways that tribes address some of the more complex issues of tribal research oversight, including jurisdiction off tribal lands and protection of individual and collective interests, to spur Native nations to create and strengthen codes as guides to use of the CARE Principles with their peoples, lands, knowledges, and resources.

The fact that most tribal citizens reside off tribal lands (Norris et al., 2012), but may participate in research, raises unique challenges to the exercise of tribal sovereignty in research. Tribes have sought to address this governance challenge by extending the application of their research codes beyond tribal lands in two situations: (1) use of materials to which tribes have a legal claim and (2) participation of tribal citizens. Some tribes extend the protection of their citizens and interests beyond their territories by linking the exercise of their sovereignty to the physical location of research materials to which they have a claim (Colorado River Indian Tribes, 2009; Gila River Indian Community, 2009; Sisseton-Wahpeton Oyate Tribe, n.d.; no date, henceforth n.d.; United Houma Nation Institutional Review Board Ordinance, n.d.; White Earth Nation, 2018; Tribal Collaboration Working Group, 2018). Other tribes address research governance challenges beyond their territories by linking the exercise of sovereignty to participation of their citizens in research, particularly in studies that implicate aspects of their tribal citizenship and affiliation in some way (Navajo Nation, 2002; Confederated Tribes of Siletz Indians, 2005; Ho-Chunk Nation, 2005; Pascua Yaqui Tribe, 2008).

Some tribal claims of ownership over specimens and data are made in the context of broader statements about tribal sovereignty. For example, the Three Affiliated Tribes (n.d.) includes a general principle of prior rights that recognizes, among other rights, “proprietary rights and interests over… all knowledge and intellectual property” associated with their resources. Similarly, the United Houma (n.d.) Institutional Review Board Ordinance codifies the rights of the Tribe, “as a self-governed and self-determined people,” to “all data and information generated and produced by… research” conducted in the community. Other codes couch the tribe’s claim to ownership of specimens and data in narrower terms (Pascua Yaqui Tribe, 2008; Confederated Tribes of Siletz Indians, 2005; Sisseton-Wahpeton Oyate Tribe, n.d.), while others stress the need for researchers to respect those claims (Mohawk Nation of Akwesasne, 1996; Cherokee Nation, 2019b).

Some codes protect not only tribal (i.e., collective) but also individual citizens’ claims to ownership and control of specimens and data (Tohono O’odham Nation, 2013; Colorado River Indian Tribes, 2009). Tribes have adopted intellectual property provisions in their codes to support individual and collective claims of ownership in specimens and data (Mohawk Nation of Akwesasne, 1996; Navajo Nation, 2002; Ho-Chunk Nation, 2005; Colorado River Indian Tribes, 2009; Turtle Mountain Band of Chippewa Indians, 2014).

Issues in research agreements pertaining to data reflect broad tribal concerns about specimens. Additional points include the need to: describe specific means of preserving confidentiality of individual and tribal data, including Assurances of Confidentiality (Mohawk Nation of Akwesasne, 1996; Ho-Chunk Nation, 2005; Gila River Indian Community, 2009); provide data disposal plans (Cherokee Nation, 2019b); and detail conditions that would allow researchers to breach their duty of confidentiality under signed agreements (Mohawk Nation of Akwesasne, 1996; Ho-Chunk Nation, 2005).

### International, Federal, and Institutional Guidelines

As institutions increasingly operationalize the CARE Principles in policy and practice, understanding how high-level principles link to tribal expectations becomes paramount. While research institutions, researchers, and funding agencies must follow appropriate federal, state, and local laws, they must also follow proper engagement and consultation procedures with tribal nations to uphold tribal law and policy pertaining to research, data, and specimens. Tribal laws and processes need to be part of robust planning and policy for research institutions and programs to implement the CARE Principles. Importantly, each Native nations’ written standards apply to research relationships with that nation only. The written standards must be balanced with ongoing community relationships to give more depth and definitions to community expectations and needs. Additionally, when no laws exist, it is the responsibility of research institutions, researchers, and funding agencies to engage in a process with participating tribal nations to obtain approvals and guidance for research and data oversight (Tribal Collaboration Working Group, 2018; National Congress of American Indians, 2019a). Finally, examining the commonalities across Native nations provides insight into broad and common ethical expectations.

### Evolving Ethical Practices

The CARE Principles, especially as indicated by tribal research codes, delineate standards for research practice. Training for researchers to understand tribal sovereignty, tribal codes, and review processes is necessary to provide the knowledge and tools to meet tribal ethical expectations. Supporting the CARE Principles requires an approach to biomedical research and policy that supports tribal ethics requirements, regardless if they have been codified as law.

Institutions, researchers, tribes, and Indigenous communities will benefit from careful attention to the CARE Principles to enhance trust and build meaningful relationships to ensure high quality translational biomedical science that emerges as tangible benefits for tribes and rural and urban Indigenous communities.

## Data Availability

The original contributions presented in the study are included in the article/Supplementary Material, further inquiries can be directed to the corresponding author.
